# The relationship between stigma and illness acceptance in type 2 diabetes and determining factors: a cross-sectional study

**DOI:** 10.1186/s12902-026-02189-y

**Published:** 2026-02-19

**Authors:** Ufuk Demirel, Fatma Hastaoglu

**Affiliations:** 1 Nursing Department, Faculty of Health Science, Zonguldak Bulent Ecevit University, Kozlu, Zonguldak, 67600 Türkiye; 2 Elderly Care Programme, Health Programmes Department, Health Services Vocational School, Sivas Cumhuriyet University, Sivas, 58140 Türkiye

**Keywords:** Acceptance of illness, Nursing, Stigma, Type 2 diabetes mellitus

## Abstract

**Background:**

Patients with type2 diabetes frequently face prejudices that result in feelings of stigma, creating an adverse feedback loop that both degrades their quality of life and complicates the management of their illness. The aim of this study is to determine the relationship between stigma and illness acceptance in type 2 diabetes mellitus and the factors that determine it.

**Methods:**

The research was conducted as a cross-sectional descriptive study and between January and June 2025 at the endocrinology clinic of an university hospital. The study included 96 patients. Data were collected using a personal information form, the Type 2 Diabetes Stigma Assessment Scale (DSAS-2), and Acceptance of Illness Scale (AIS). The data were analysed using IBM SPSS 25.0 software. A STROBE checklist was the reporting guide for this study.

**Results:**

Participants’ DSAS-2 and AIS total mean scores were 46.56 ± 14.44 and 22.93 ± 6.25, respectively. A positive correlation has been determined between DSAS-2 and AIS scores. (*R* = 0.560, *p* < 0.001). Participants who had diabetes for 6–11 years, received combination therapy (insulin and oral antidiabetic agents), were hospitalised due to DM in the past year, and required assistance with care had higher DSAS-2 and AIS scores compared to others (*p* < 0.05). Multiple regression analysis showed that the type of diabetes treatment was a significant determinant of DSAS-2 and AIS scores.

**Conclusion:**

Patients receiving combination therapy demonstrated significantly higher stigma scores and greater illness acceptance compared to other treatments. The correlation between stigma and acceptance of illness is positive. Authors suggest that acceptance of illness should not always be perceived as having a positive effect. It is strongly recommended to incorporate stigma screening into the management plan of patients requiring both insulin and oral antidiabetic drugs. A comprehensive evaluation of patients with diabetes must account for both stigma and illness acceptance concurrently.

**Clinical trial number:**

Not applicable.

## Introduction

Diabetes mellitus (DM) is among the leading causes of mortality worldwide, with approximately one million deaths annually directly attributable to DM. The International Diabetes Federation (IDF) predicts that 589 million people worldwide will have diabetes by 2025 [1]. Turkey, one of the 60 countries in the IDF European Region, has been reported to have a notably high diabetes prevalence of 16.3% [1].

Diabetes-related stigma is defined as the experience of negative emotions, such as social exclusion, rejection or blame, due to perceived discrimination associated with diabetes [2]. Among adults with type 2 diabetes mellitus (T2DM), stigma prevalence ranges from 12% to 70% [3]. Recognisable features of diabetes, including insulin injections, blood glucose monitoring, dietary restrictions, obesity and hypoglycaemic episodes, contribute to increased stigma in these individuals [2]. Diabetes stigma is associated not only with sociodemographic factors such as age, marital status, and education level, but also with insulin use and daily insulin frequency [4, 5]. Zhang et al.‘s study identified diabetes complications, insulin use, diabetes duration and monthly income as significant factors related to stigma [6]. A meta-analysis by Akyirem et al. demonstrated a positive correlation between HbA1c levels and stigma [7]. Taken together, these findings suggest that diabetes stigma may lead to adverse health outcomes.

The development of adverse health outcomes related to diabetes involves numerous psychosocial and behavioural factors [8]. Disease acceptance refers to an individual’s ability to acknowledge their illness, emotionally come to terms with the diagnosis, and functionally incorporate it into daily life. In individuals who can not accept their illness are more likely to exhibit negative behaviours such as reduced compliance with treatment, inadequate self-care and an increased psychological burden [9]. Arı and Özdelikara found significant negative correlations between disease acceptance and HbA1c, postprandial blood glucose, and fasting blood glucose levels [10]. Similarly, Seo’s study demonstrated that disease acceptance mediates the relationship between self-stigma and quality of life in individuals with T2DM [11]. Current evidence indicates that disease acceptance among individuals with T2DM is frequently inadequate. Khazew and Faraj reported suboptimal disease acceptance levels in patients with T2DM, whereas Liu et al. observed moderate acceptance levels [12, 13].

Multiple factors influence both diabetes-related stigma and disease acceptance in individuals with T2DM. However, there are very few studies in the literature that address these two variables together. Therefore, this cross-sectional study aimed to: (1) investigate the association between diabetes-related stigma and disease acceptance, and (2) identify factors associated with both diabetes-related stigma and disease acceptance in individuals with T2DM. It is anticipated that the study findings will contribute to a strong evidence base, supporting nurses in providing holistic care to people with diabetes. Additionally, identifying the determinants of stigma and illness acceptance will enable nurses to detect at-risk individuals early and plan appropriate nursing interventions.

## Methods

### Study design

The research was conducted as a cross-sectional descriptive study. The research design was selected as it was the most appropriate method for the study’s primary aim of measuring the relationship between stigma and illness acceptance at a single point in time. This design was chosen for its suitability for relational analysis, practical efficiency, and its ability to provide pioneering evidence on this relationship in the literature. This study was conducted in Endocrinology Clinic of a university hospital in Sivas Cumhuriyet University between January and June 2025. The research data was collected via face-to-face interviews using a personal information form, the type 2 diabetes mellitus stigma assessment scale, and the acceptance of illness scale.

### Sample and setting

Patients hospitalized in the Endocrinology Clinic of a university hospital in Sivas Cumhuriyet University constituted the population of the study. The study population consisted of patients hospitalised at the Endocrinology Clinic of a university hospital in Turkey. This study was conducted in a university hospital. The primary reason for this selection was the necessity to effectively reach the target population of individuals with T2DM. Unlike other hospitals in the city, the chosen university hospital features a full-fledged endocrinology clinic, which serves as a central hub for a large number of diabetic patients receiving regular follow-up and treatment. This setting enabled the recruitment of the required sample size and provided access to a heterogeneous patient population (with variations in age, gender, disease duration, and complications). Furthermore, as university hospitals typically manage more complex and advanced cases, this choice allowed us to study a patient group in which psychosocial dimensions such as stigma and illness acceptance might be more pronounced. Using sampling calculations for unknown population and referencing the 13.7% diabetes prevalence reported by the Turkish Society of Endocrinology and Metabolism [14], we determined that a minimum of 93 participants were required for the study (95% confidence interval, 5% margin of error). The study included patients who: (1) were over 18 years of age, (2) had received a T2DM diagnosis at least six months prior, (3) were taking oral antidiabetic agents and/or insulin, and (4) signed informed consent. Exclusion criteria from the study were: (1) documented cognitive impairment in medical records, (2) any diagnosed psychiatric disorder, or (3) comorbid conditions known to influence stigma perception (e.g., epilepsy, HIV/AIDS, limb amputation, or active infectious diseases). An a priori power analysis was conducted using the G*Power software. Based on an expected medium effect size (Cohen’s f² = 0.15), a statistical power of 0.80, and an alpha level of 0.05, the minimum required sample size was calculated as 93 participants. The study was completed with 96 individuals with Type 2 Diabetes Mellitus, thus fulfilling the minimum required sample size for sufficient statistical power.

### Data collection

Data were collected using a personal information form, the type 2 diabetes mellitus stigma assessment scale, and the acceptance of illness scale. The Stigma in Diabetes Scale for Type 2 Diabetes was originally developed by Liu et al. [15] and the Illness Acceptance Scale by Felton et al. [16]. In this study, the validated Turkish versions of these scales were used. The validity and reliability information for the scales are provided below in the descriptions of each respective instrument.

#### Personal information form

This 14-question form was developed by researchers based on literature reviews [17, 18] to collect participants’ sociodemographic data (age, sex, education status etc.)and information on certain medical conditions.

#### The stigma in diabetes scale for type 2 diabetes (DSAS-2)

The scale was developed by Browne et al. [19] The Turkish validity and reliability study of the scale was conducted by İnkaya and Karadağ in 2021 [20]. The scale consists of three sub-dimensions: treated differently (6 items; items 1, 4, 7, 10, 14, and 17), blame and judgment (7 items; items 2, 3, 5, 8, 12, 16, 19), and self-stigma (6 items; 6, 9, 11, 13, 15, 18) and consists of 19 items. The scale is a five-point Likert scale. The scale score ranges from 19 to 95 points. A higher score indicates a higher level of stigma. The Cronbach’s alpha coefficient was found to be 0.92.^18^ In this study, the Cronbach’s alpha coefficient of the scale was found to be 0.94.

#### Acceptance of illness scale (AIS)

The Acceptance of Illness Scale was developed by Felton and Revenson in the United States in 1984 [21]. The Turkish validity and reliability study was conducted by Büyükkara Besen in 2011 [21]. The scale consists of eight items and is a five-point Likert scale. The scale score ranges from 8 to 40 points and is a general measure of illness acceptance. A high score is evidence of acceptance of the illness and indicates the absence of negative feelings about the illness and the presence of acceptance. The Cronbach’s alpha coefficient of the scale is 0.79 [20]. In this study, the Cronbach’s alpha coefficient of the scale was found to be 0.85.

#### Statistical analysis

The data were analyzed using the IBM SPSS version 25.0 (IBM Inc., Armonk, NY, USA). In the statistical analysis of the data, when comparing two independent groups, the student-t test was used to analyse the significance of the difference between two means when the parametric test assumptions were met, and the Mann-Whitney U test was used to analyse the significance of the difference between two means that did not show a normal distribution. For three or more variables, the One-Way ANOVA test was used when the assumptions of parametric tests were met, and the Kruskal-Wallis test was used when the assumptions of parametric tests could not be met. The relationship between scale scores and age, as well as between scale scores, was evaluated using Pearson correlation analysis, with a significance level of 0.05. Simple regression analysis was used to assess the relationship between scale scores, and multiple linear regression analysis was used to identify the factors influencing scale scores.

## Results

The participants’, 69.8% were female, 88.5% were married, 43.8% had completed primary school, 79.2% were of moderate economic status and 88.5% were not in active employment. Of participants 43.8% were obese. The average age of participants was 61.19 ± 16.33 (Table 1).

**Table 1:** Participants’ socio-demographic characteristics and distribution of scale scores according to the characteristics (*n* = 96)

		*n* (%)	Treated Differently Mean ± SD			Blame and Judgment Mean ± SD			Self- Stigma Mean ± SD		DSAS-2 Total Mean ± SD		AIS Mean ± SD	
**Gender**														
Female		67 (69.8)	14.86 ± 4.50			18.16 ± 5.49			11.50 ± 4.15		46.68 ± 14.09		23.10 ± 5.77	
Male		29(30. 2)	15.34 ± 5.55			17.65 ± 5.69			11.00 ± 4.94		46.27 ± 15.48		22.55 ± 7.34	
T			-0.445			0.412			0.518		0.127		0.063	
P			0.657			0.681			0.605		0.899		0.693	
**Marital Status**														
Single		11 (11.5)	15.54 ± 5.61			17.27 ± 6.16			12.27 ± 4.73		47.54 ± 16.81		24.09 ± 8.83	
Married		85 (88.5)	14.94 ± 4.74			18.01 ± 5.47			11.23 ± 4.35		46.43 ± 14.22		22.78 ± 5.89	
U			-0.664			-0.473			-0.951		-0.483		-0.593	
P			0.507			0.636			0.341		0.629		0.553	
**Education Level**														
Illiterate		21 (21,9)	14.14 ± 3.70			16.71 ± 4.18			10.66 ± 12.61		43.47 ± 10.41		23.19 ± 6.02	
Literate		18 (18.8)	15.00 ± 3.89			19.11 ± 5.02			12.61 ± 3.94		49.38 ± 12.62		25.11 ± 6.59	
Primary School		42 (43.8)	15.35 ± 4.92			18.09 ± 5.31			11.19 ± 4.47		46.73 ± 14.04		22.97 ± 5.89	
High School		9 (9.4)	15.44 ± 7.03			18.22 ± 8.36			11.66 ± 6.70		47.44 ± 22.65		20.11 ± 7.45	
Bachelor’s		6 (6.3)	15.00 ± 7.21			18.33 ± 8.35			10.66 ± 5.60		46.33 ± 21.97		19.50 ± 5.54	
F			0.235			0.467			0.551		0.412		1.495	
P			0.918			0.760			0.699		0.800		0.210	
**Economik status**														
Poor		9 (9.4)	18.22 ± 4.84			19.77 ± 5.44			13.11 ± 6.05		53.55 ± 16.07		27.55 ± 7.66	
Moderate		76 (79.2)	14.57 ± 4.41			17.86 ± 5.29			11.21 ± 4.05		45.80 ± 13.50		22.81 ± 5.86	
Good		11 (11.5)	15.36 ± 6.63			17.54 ± 7.31			10.90 ± 5.20		46.09 ± 18.98		20.00 ± 6.118	
F			2.401			0.518			0.816		1.169		3.905	
P			0.096			0.597			0.445		0.315		**0.024**	
**Body Mass Index**														
Normal		11 (11.5)	16.18 ± 4.62			17.27 ± 4.67			11.45 ± 3.85		47.75 ± 11.81		22.27 ± 7.26	
Overweight		37 (38.5)	15.05 ± 5.72			18.21 ± 6.64			11.67 ± 5.45		47.05 ± 17.89		23.83 ± 6.89	
Obese		42 (43.8)	14.64 ± 4.32			17.88 ± 5.02			11.02 ± 3.71		45.78 ± 12.74		22.07 ± 5.55	
Morbidly obese		6 (6.2)	15.16 ± 2.40			18.16 ± 3.43			11.50 ± 2.58		47.33 ± 6.28		24.66 ± 5.08	
F			0.294			0.035			0.146		0.072		0.711	
P			0.829			0.991			0.932		0.975		0.548	
**Working Status**														
Not working		85 (88.5)	15.44 ± 4.55			18.48 ± 5.21			11.83 ± 4.32		48.02 ± 13.86		23.845.88	
Working		11 (11.5)	11.63 ± 5.66			14.36 ± 6.77			7.63 ± 2.90		35.27 ± 14.47		15.90 ± 4.45	
U			-2.216			-2.133			-3.068		-2.595		-3.885	
P			**0.027**			**0.033**			**0.002**		**0.009**		**< 0.001**	
		**Mean ± SD** **(Min.-Max.)**	**r**		**p**	**r**	**p**		**r**	**p**	**r**	**p**	**r**	**p**
**Age**		61.19 ± 16.33 (20–97)	0.157		0.127	0.200	0.051		0.209^*^	**0.041**	0.202*	**0.049**	0.416**	**< 0.001**

* Correlation is significant at the 0.05 level. ** Correlation is significant at the 0.01 level

A statistically significant difference didn’t found between the mean DSAS-2 and AIS scores participants’age, gender, educational status, or body mass index (*p* > 0.05). DSAS-2 scores did not differ significantly based on their economic status (*p* > 0.05), although AIS score was statistically different according to economic status (*p* < 0.05). Those who were not actively working had higher DSAS-2 subscale and total scores and AIS scores (*p* < 0.05) (Table 1).

There was a weak positive correlation between age and DSAS-2 score and the self-stigma subscale score (*r* = 0.202, *p* = 0.049; *r* = 209, *p* = 0.042, respectively). Additionally, there was a moderate positive correlation between age and the AIS score (*r* = 0.416, *p* < 0.001) (Table 1).

Table 2 show the participants’ 32.3% had been diagnosed with diabetes for 1–5 years. Almost equal proportions of patients received oral antidiabetic and insulin for diabetes treatment (40.6% and 39.6%, respectively). Participants’ 68.8% reported no diabetes-related hospitalizations in the during the previous year, while 89.6% indicated they attending regular diabetes follow-ups. Of participants 45.8% required assistance with care. DSAS-2 and AIS scores showed significant variations based on diabetes duration, treatment modality, daily insulin dosage, diabetes-related hospitalizations in the preceding year, and requirement for care assistance (*p* < 0.05). However, neither comorbid conditions nor adherence to regular diabetes follow-ups demonstrated significant associations with these scale scores(*p* > 0.05).The participants’ mean DSAS-2 score was 46.56 (± 14.44), and their mean AIS score was 22.93 (± 6.25). The mean scores for the DSAS-2 subscales are presented in Table 3.

**Table 2:** Distribution of participants’ disease-related information and corresponding scale scores (*n* = 96)

	*n* (%)	Treated Differently Mean ± SD	Blame and Judgment Mean ± SD	Self- Stigma Mean ± SD	DSAS-2 Total Mean ± SD	AIS Mean ± SD
**DM duration**						
< 1 year^1^	18 (18.8)	13.33 ± 5.00	15.88 ± 5.83	10.22 ± 3.54	41.55 ± 14.47	20.38 ± 5.69
1–5 years^2^	31 (32.3)	14.41 ± 4.58	18.19 ± 5.58	10.48 ± 3.77	45.29 ± 13.39	21.61 ± 6.52
6–11 years^3^	28 (29.2)	17.35 ± 4.75	20.25 ± 5.50	13.75 ± 4.91	53.96 ± 14.96	25.64 ± 6.00
12 years and above^4^	19 (19.8)	14.10 ± 4.13	16.42 ± 4.07	10.31 ± 4.12	42.47 ± 11.90	23.52 ± 5.45
F		3.589	3.149	4.365 3 > 1	4.128 3 > 1,4	3.520 3 > 1
P		**0.017**	**0.029**	**0.006**	**0.009**	**0.018**
**Type of treatment**						
Oral antidiabetic^1^	39 (40.6)	13.25 ± 4.81	16.23 ± 5.76	9.82 ± 4.19	41.28 ± 14.87	19.53 ± 5.06
İnsülin^2^	38 (39.6)	15.44 ± 4.16	18.65 ± 5.08	11.18 ± 3.19	47.47 ± 11.46	23.57 ± 5.92
Oral antidiabetic ve insulin^3^	19 (19.8)	17.73 ± 4.81	20.36 ± 4.93	14.84 ± 5.04	55.57 ± 14.72	28.63 ± 4.47
F		6.440 3 > 1	4.285 3 > 1	10.017 3 > 1,2	7.213 3 > 1	19.096 3 > 1,2 2 > 1
P		**0.002**	**0.017**	**< 0.001**	**0.001**	**< 0.001**
**Number of insulin injections per day**						
Not using ^1^	39 (40.6)	13.02 ± 4.72	16.00 ± 5.77	9.37 ± 4.00	40.32 ± 14.36	18.86.5.18
Once ^2^	15 (15.6)	17.06 ± 5.74	19.62 ± 6.15	14.06 ± 9.37	53.37 ± 16.70	25.81 ± 6.84
Twice ^3^	23 (23.9)	16.41 ± 4.15	19.91 ± 4.68	12.54 ± 4.28	51.12 ± 12.49	25.04 ± 5.80
Three times ^4^	12 (12.5)	15.50 ± 3.98	17.66 ± 4.99	11.50 ± 3.63	47.00 ± 12.41	27.00 ± 3.78
Four times ^5^	7 (7.3)	15.14 ± 3.43	19.00 ± 3.46	11.28 ± 1.11	47.57 ± 6.34	23.71 ± 3.19
F		3.083 3 > 1	2.491 3 > 1	4.394 2 > 1	3.571 3 > 1	9.008 4,3,2 > 1
P		**0.020**	**0.049**	**0.003**	**0.009**	**< 0.001**
**Comorbidity**						
Yes	70 (72.9)	15.44 ± 4.73	18.35 ± 5.34	11.72 ± 4.53	47.75 ± 14.29	23.65 ± 6.25
No	26 (27.1)	13.84 ± 4.93	17.07 ± 5.99	10.34 ± 3.84	43.34 ± 14.65	21.00 ± 5.94
T		1.451	1.008	1.379	1.335	1.874
P		0.150	0.316	0.171	0.185	0.064
**Hospitalization due to DM in the past year**						
Yes	30 (31.3)	16.66 ± 4.94	19.66 ± 5.55	13.23 ± 4.91	52.06 ± 15.35	26.66 ± 5.60
No	66 (68.8)	14.25 ± 4.60	17.25 ± 5.39	10.50 ± 3.87	44.06 ± 13.40	21.24 ± 5.81
T		2.322	2.010	0.199	2.591	4.282
P		**0.022**	**0.047**	**0.004**	**0.011**	**< 0.001**
**Regular DM monitoring**						
Yes	86 (89.6)	15.05 ± 4.85	18.08 ± 5.52	11.39 ± 4.48	46.66 ± 14.60	22.69 ± 5.95
No	10 (10.4)	14.60 ± 4.76	17.40 ± 5.87	11.00 ± 3.62	45.70 ± 13.74	25.00 ± 8.52
Z		0.908	0.708	0.584	-0.144	-0.943
P		0.778	0.714	0.789	0.885	0.346
**Need for assistance with care**						
Yes	44 (45.8)	16.45 ± 4.70	19.22 ± 4.86	12.95 ± 4.80	51.00 ± 14.18	26.22 ± 5.43
No	52 (54.2)	13.78 ± 4.61	16.98 ± 5.88	10.00 ± 3.50	42.80 ± 13.70	20.15 ± 5.54
T		2.796	2.015	3.386	2.871	5.396
P		**0.006**	**0.047**	**0.001**	**0.005**	**< 0.001**

**Table 3:** DSAS and AIS scale scores

	Min.-Max.	Mean ± SD
Treated differently	6–27	15.01 ± 4.81
Blame and Judgment	7–33	18.01 ± 5.52
Self- Stigma	5–25	11.35 ± 4.38
DSAS-2 Total Score	19–95	46.56 ± 14.44
AIS Total Score	8–37	22.93 ± 6.25

Regression analysis revealed a significant positive association between DSAS-2 and AIS scores (β = 0.56, *p* < 0.001). However, the model’s explanatory power is limited (R²=0.314) (Table 4).

**Table 4:** Simple linear regression analysis of DSAS total score on AIS score

	B	SE	β	t	*p*
Constant	11.650	1.803	-	6.462	< 0.001
AIS toplam	0.242	0.037	0.560	6.552	< 0.001

1. *R* = 0.560, R^2^ = 0.314, Adj R^2^ = 0.306, F:42.933; *p* < 0.001 (Adj: Adjusted R Square)

Multiple linear regression analysis was used to identify the factors affecting the DSAS-2 score. The analyses revealed that the method of diabetes treatment was a significant determinant of DSAS-2 scores. Combination therapy comprising both insulin and oral antidiabetic agents emerged as a statistically significant independent predictor in the regression model (R²=0.203) (Table 5).Table 6 shows the results of regression analysis regarding the AIS scores of the participants. Active working life, diabetes treatment method, and need for assistance with care are statistically significant determinants of the AIS score. According to this result, individuals who are not working, who use oral antidiabetic drugs and insulin together for the treatment of diabetes, and who need assistance with care have a higher AIS score.

**Table 5:** Multiple regression analysis: factors affecting DSAS-2 scores

	B	SE	β	t	*p*
Constant	19.914	9.672	-	2.509	0.042
Age	0.016	0.105	0.019	0.156	0.877
Working Status	-7.589	4.754	-0.168	-0.1598	0.114
DM Duration	-0.173	1.563	-0.012	-0.111	0.912
Type of treatment	5.712	2.482	0.298	2.301	**0.024**
Number of insulin injections per day	-0.687	1.436	-0.062	-0.479	0.633
Hospitalization due to DM in the past year	2.801	3.323	0.090	0.843	0.402
Need for assistance with care	3.339	3.344	0.118	1.016	0.312

2. *R* = 0.450, R^2^ = 0.203, Adj. R2 = 0.139, F = 3.194, *p* = 0.005

**Table 6:** Multiple regression analysis: factors affecting AIS score

	B	SE	β	t	*p*
Constant	8.901	4.405	-	2.020	0.046
Age	0.053	0.037	0.138	1.435	0.155
Working status	-3.575	1.653	-0.183	-2.162	**0.033**
Economic status	-1.272	1.125	-0.093	-1.130	0.261
DM duration	0.011	0.543	0.002	0.21	0.983
Type of treatment	2.529	0.873	0.304	2.898	**0.005**
Number of insulin injections per day	0.363	0.500	0.076	0.725	0.470
Hospitalization due to DM in the past year	1.273	1.193	0.095	1.067	0.289
Need for assistance with care	2.609	1.167	0.209	2.235	**0.028**

3. *R* = 0.701, R^2^ = 0.492, Adj R^2^ = 0.445, F:10.524; *p* < 0.001

## Discussion

This study investigated the factors influencing stigma perception and illness acceptance in individuals with type 2 diabetes mellitus (T2DM), while also examining the interrelationship between these constructs. The subsequent discussion interprets these findings in relation to existing literature.

There was a weak significant positive correlation between age and total DSAS-2 scores. A similar study found no correlation between age and DSAS-2 score [5]. In multiple linear regression analysis, the significance of the independent effect of age was lost when factors such as employment status, duration of diabetes diagnosis and duration of diabetes treatment were added to the model. This finding indicates that the relationship between age and stigma is not exclusively age-dependent. This result is similar to the literatüre [22]. This study revealed a statistically significant, moderate positive correlation between age and AIS scores. These findings suggest that individuals become more accepting of the disease as they age. However, Khazew and Faraj reported that no correlation exists between age and AIS score. The observed discrepancy between studies may be attributable to cultural variations in illness perception and adaptation processes.

The analysis revealed no significant associations between DSAS-2 and AIS total scores and gender, education level, or BMI. However, AIS scores varied significantly according to economic status. Participants who reported poorer economic status demonstrated significantly higher AIS scores. However, Can et al. found that individuals with higher socioeconomic status had significantly higher AIS scores [23]. Although participants reporting lower socioeconomic status demonstrated elevated AIS scores, this association should not be interpreted as reflecting better disease management [7]. This acceptance should not be construed as necessarily reflecting conscious or adaptive psychological processes; it may alternatively represent passive resignation or learned helplessness [24].

The present study found significantly higher DSAS-2 total scores among unemployed participants compared to their employed participants. The workplace is a critical setting for social interaction and solidarity. The attitude in which colleagues at work understand the condition can have a significant impact on the perceived stigma experienced by employees with diabetes. Individuals who are not in gainful employment may find themselves deprived of this support mechanism and consequently become more susceptible to feelings of isolation and exclusion. The attitude in which colleagues at work understand the condition can have a significant impact on the perceived stigma experienced by employees with diabetes. Olesen et al. reported that only 6% of individuals with type 2 diabetes experienced workplace discrimination in their study [25]. However, when the effects of overweight/obesity were controlled for, it was reported that the stigma disappeared. Consequently, the workplace itself does not inherently constitute a source of stigma [25].

The analysis revealed employment status to be a statistically significant predictor of AIS scores. This outcome may be attributable to the greater feasibility of implementing diabetes management protocols in domestic environments. Although unemployed individuals may demonstrate better diabetes acceptance, they might experience greater stigma due to lack of social support. In contrast to our findings, Turen et al. reported significantly higher AIS scores among employed individuals in their study [26]. The workplace’s role in either enabling or impeding disease management could significantly influence illness acceptance levels.

A significant relationship was found between diabetes duration and DSAS-2 total score, self-stigma subscale score, and AIS score. However, this relationship is internally inconsistent. Thus, we cannot conclusively state that ‘as years since diabetes diagnosis increases, stigma and/or acceptance necessarily increases”. The highest DSAS-2 total scores were observed among patients who had diaetes for 6–11 years. Newly diagnosed individuals have lower DSAS-2 total and self-stigma scores than those who have had diabetes for 6–11 years. Self-stigma, or internalized stigma, occurs when individuals with diabetes adopt society’s negative stereotypes or discriminatory attitudes, leading them to perceive themselves as inadequate, shameful, or unworthy. As the duration of diabetes increases, individuals may face greater exposure to negative societal attitudes. This, in turn, may lead to higher scores on the DSAS-2 and its self-stigma subscale. The lower DSAS-2 total scores observed in individuals with 12 or more years of diabetes may suggest that coping with diabetes-related stigma requires an extended period of time. This finding is consistent with the study by Özkan Tuncay and Koçyiğit involving individuals with type 2 diabetes [22]. This finding is consistent with the study by Zhang et al., which reported lower stigma levels in the group with the longest disease duration [6].

In this study, the relationship between diabetes duration and AIS score is shown to be parallel to that between diabetes duration and DSAS-2 total score. While there appears to be an increase in acceptance of the disease with an increase in duration of diabetes, a marked decline is observed in patients with a diagnosis duration of 12 years or more. Consequently, the experience of long-term illness has the potential to impede the acceptance process by giving rise to challenges such as the struggle to cope with chronic conditions and the development of exhaustion over time. Turen et al. found in their study that AIS scores were lower among people with diabetes for a duration of 16 years or more [26]. This result is consistent with our research findings. However, a contrary finding was reported in another study, which found no association between diabetes duration and AIS score [12]. The observed discrepancy in research outcomes may be attributable to socio-demographic variations within the study sample, the presence of complications, and other contributing factors.

The present study has demonstrated that the type of diabetes treatment received by patients was found to be a significant determinant of DSAS-2 total score. The findings of this study demonstrated that patients who were administered both insulin and oral antidiabetic treatment exhibited higher DSAS-2 total scores. Research has indicated that the utilisation of insulin among individuals diagnosed with type 2 DM is a substantial contributing factor to the experience of stigma [5, 6].

In addition, type of diabetes treatment was also found to be a determinant of AIS score in this study. It can be hypothesised that combination therapy may enhance disease acceptance, but that this treatment approach could potentially lead to increased stigma over time. In this context, high acceptance may not always be an indicator of a positive psychological state [27]. Even if an individual has accepted their illness, this situation may cause them to begin to identify themselves with the label of ‘sick.’ This coincides with internalised stigma [11].

The study found that individuals who had been hospitalised due to DM in the past year had higher DSAS-2 total scores than other subjects. Stigma can adversely affect physical health by limiting access to healthcare services and reducing treatment adherence, potentially leading to problems that require hospitalization [28]. Conversely, hospitalisation due to the present health problem may have exacerbated the level of stigma, engendering perceptions such as inadequacy and self-blame. The relationship between stigma and hospitalization appears to be bidirectional. Aslan et al. found that unplanned hospitalisation in the past year increased stigma [5].

In this study, AIS scores were found to be higher in patients admitted to hospital in the last year. Acceptance of the illness may indicate the patient’s satisfaction with their current state, potentially reducing motivation for further health-improving efforts [12]. Consequently, a high AIS score may not prevent hospitalization, as it could reflect passive resignation rather than active disease management.

In this study, a higher mean DSAS-2 total score was observed in patients requiring care compared to those not requiring care. Zheng et al. found that diabetic individuals who were completely dependent on self-care had higher stigma scores than those who were not dependent [27]. Cho et al. found a negative correlation between self-care behaviour and stigma [29]. The results of the study are similar. The necessity for care assistance may be associated with variables such as physical disabilities, comorbidity, or protracted illness duration [30]. This predicament may result in patients encountering elevated levels of stigma.

The present study found that the need for care assistance was a significant predictor of disease acceptance. The study show that the acceptance levels of the disease among individuals requiring care assistance were higher than those not in need of such assistance. This result can be interpreted as a combination of different dimensions. Individuals who accept their illness may be more willing to seek help. Alternatively, the caregiver’s adoption of a normalising stance may promote acceptance [31].

The participants’ DSAS-2 different behoviours subscale, blame and judgement subscale, self-stigmatization subscale score, and DSAS-2 total score were as follows: 15.01 ± 4.81, 18.01 ± 5.52, 11.35 ± 4.38, and 46.56 ± 14.44, respectively. Özkan Tuncay and Koçyiğit found the total DSAS-2 total score to be 47.85 ± 17.81, while Aslan et al. found the total DSAS-2 total score in insulin-using patients with T2DM to be 57.74 ± 11.13 [5, 22]. Xing et al. found the DSAS-2 total score to be 41.74 ± 12.57 [8]. In Li et al.‘s study, the mean total score on the DSAS-2 was reported as 54.30 ± 12.22 [32]. Research findings show that individuals with type 2 DM experience stigma at different levels. In this study, a significant positive correlation was found between the DSAS-2 total score and the AIS score (β = 0.56, *p* < 0.001). However, the low explanatory power of the model (R² = 0.314) indicates the complex nature of this relationship. The findings of this study indicate the possibility that unmeasured third variables (e.g., disease awareness, social support, depression, or self-care) may be driving the observed positive relationship. A limitation of the present study is the absence of measurement of these factors.

Conversely, individuals may disclose their chronic conditions to those around them as a result of accepting their illness. Nevertheless, uncertainty may prevail with regard to the extent of information to be disclosed and the relevant recipients. The consequences of such experiences may include rejection and stigmatisation, difficulties in coping with the reactions of others, and a sense of loss of control [33]. The limited number of research examining the relationship between stigma and acceptance of illness in individuals with T2DM [11] is a significant gap in the literature. When studies on the subject are examined, a study on individuals with psoriasis revealed a significant positive correlation between acceptance of illness and stigmatisation [34]. These findings are consistent with our results. This study addresses a critical gap in the literature by examining the understudied relationship between stigma and illness acceptance, thereby making a significant contribution to the field.

In this study, the AIS score was 22.93 ± 6.25. Özyalçın and Sanlier found the AIS scores of individuals with T2DM to be 30.2 ± 5.62 [35]. In another study conducted in Turkey, the AIS score was 27.06 ± 9.57.^25^ In a study conducted by Rogon et al. in Poland, the majority of individuals with T2DM were found to have a low level of acceptance of their disease [36]. A study conducted in China found that the AIS score for individuals with Type 2 DM was 24.50 ± 7.34 [13]. Consistent with the findings, a previous research involving individuals with type 2 diabetes mellitus (T2DM) reported comparable results [37]. The results of the study are consistent with previous studies and show that individuals with diabetes do not accept their disease well. While cultural differences exist, the inherent complexity of diabetes management and the chronic nature of the condition appear to similarly influence illness acceptance across populations. Furthermore, these findings suggest that integrating the ‘assessment and enhancement of illness acceptance’ as a fundamental component into diabetes care standards may be a universal requirement. Specifically, the implementation of counseling and educational programs aimed at improving illness acceptance, particularly in the post-diagnosis period, is recommended.

### Study limitations

The results of this study are limited to this sample group. A limitation of this study is that it does not consider variables that may influence these concepts in order to understand the relationship between stigma and acceptance of illness. Future researchs is recommended that includes dependent variables that may influence stigma and disease acceptance.

## Conclusions

In this study, stigma was found to be higher in those using insulin and oral antidiabetics for diabetes treatment than in others. Our analysis identified three critical factors influencing disease acceptance: (1) employment status, (2) type of diabetes treatment, and (3) degree of care dependency. A positive correlation was found between DSAS-2 and AIS total scores. While the acceptance of the disease can act as a form of protection, it can also become a dynamic that feeds stigma when it leads to a narrowing of social and identity perceptions. It is recommended that future studies encompass third variables that may have a bearing on both stigma and acceptance of the disease.

A striking finding of our study is the observed positive correlation between illness acceptance and perceived stigma, which contrasts with the expected inverse relationship. This challenges the prevalent assumption that “acceptance is invariably positive” and reveals that the association between these two constructs is more complex than previously thought. This unexpected relationship may be interpreted as a manifestation of internalized stigma, whereby greater illness acceptance could reflect a form of resignation to the disease’s social label rather than a healthy psychological adaptation. Therefore, in clinical practice, illness acceptance should not be automatically regarded as a favorable outcome; its nature and interaction with stigma must be carefully evaluated. In this context, our findings underscore the necessity for holistic care interventions in diabetes management.

## Data Availability

Data for this study were obtained from patients in the endocrinology clinic of a university hospital. Participants were given verbal and written statements stating that the data would be used solely for research purposes and would not be shared with anyone else. Due to research ethics guidelines, sharing the data is not appropriate.The Relationship Between Stigma and Illness Acceptance in Type 2 Diabetes and Determining Factors: A Cross-Sectional Study.

## References

[1] International Diabetes Federation (IDF). IDF Diabetes Atlas, 11th edition. Brussels, Belgium: International Diabetes Federation. 2025. Accessed: June 9, 2025. http://www.diabetesatlas.org.

[2] Embick R, Jackson M, Stewart R. The impact of stigma on the management of type 1 diabetes: A systematic review. Diabet Med. 2024;41(4):e15299. 10.1111/dme.15299. 38361327 10.1111/dme.15299

[3] Eitel KB, Roberts AJ, D’Agostino R, et al. Diabetes stigma and clinical outcomes in adolescents and young adults: the SEARCH for diabetes in youth study. Diabetes Care. 2023;46(4):811–8. 10.2337/dc22-1749. 36883290 10.2337/dc22-1749 PMC10090897

[4] Taher TMJ, Ahmed HA, Abutiheen AA, Alfadhul SA, Ghazi HF. Stigma perception and determinants among patients with type 2 diabetes mellitus in Iraq. J Egypt Public Health Assoc. 2023;98(1):20. 10.1186/s42506-023-00145-5. 38017311 10.1186/s42506-023-00145-5 PMC10684431

[5] Aslan EÖ, Toygar İ, Feyizoğlu G, Polat S, Eti Aslan F. Relationship between the insulin use and stigma in type 2 diabetes mellitus. Prim Care Diabetes. 2023;17(4):373–8. 10.1016/j.pcd.2023.05.002. 37217393 10.1016/j.pcd.2023.05.002

[6] Zhang YB, Yang Z, Zhang HJ, Xu CQ, Liu T. The role of resilience in diabetes stigma among young and middle-aged patients with type 2 diabetes. Nurs Open. 2023;10(3):1776–84. 10.1002/nop2.1436. 36289558 10.1002/nop2.1436 PMC9912450

[7] Akyirem S, Ekpor E, Namumbejja Abwoye D, Batten J, Nelson LE. Type 2 diabetes stigma and its association with clinical, psychological, and behavioral outcomes: A systematic review and meta-analysis. Diabetes Res Clin Pract. 2023;202:110774. 10.1016/j.diabres.2023.110774. 37307898 10.1016/j.diabres.2023.110774

[8] Xing S, Liu Y, Zhang H, Li B, Jiang X. The mediating role of diabetes stigma and self-efficacy in relieving diabetes distress among patients with type 2 diabetes mellitus: a multicenter cross-sectional study. Front Psychol. 2023;14:1147101. 10.3389/fpsyg.2023.1147101. 37575426 10.3389/fpsyg.2023.1147101 PMC10416640

[9] Wang RH, Chen SY, Lee CM, Lu CH, Hsu HC. Resilience, self-efficacy and diabetes distress on self-management behaviours in patients newly diagnosed with type 2 diabetes: A moderated mediation analysis. J Adv Nurs. 2023;79(1):215–22. 10.1111/jan.15483. 36317455 10.1111/jan.15483

[10] Arı N, Özdelikara A. The effect of family support on acceptance and treatment adaptation in type 2 diabetes patients applied to internal medicine clinics: Ordu Province sample. Turk J Diab Obes. 2022;6(1):39–48. 10.25048/tudod.1018441.

[11] Seo K. The mediating role of acceptance action and self-care in diabetes self-stigma’s impact on type 2 diabetes quality of life: A cross-sectional study. Behav Sci (Basel). 2023;13(12):993. 10.3390/bs13120993. 38131849 10.3390/bs13120993 PMC10740683

[12] Khazew HR, Faraj RK. Illness acceptance and its relationship to health-behaviors among patients with type 2 diabetes: A mediating role of self-hardiness. Curr Probl Cardiol. 2024;49(8):102606. 10.1016/j.cpcardiol.2024.102606. 38723795 10.1016/j.cpcardiol.2024.102606

[13] Liu X, Zhang Q, Huang L, Zhuang Y. Associations between disease acceptance and dietary adherence in patients with type 2 diabetes mellitus in china: A cross-sectional study. Diabetes Res Clin Pract. 2025;224:112196. 10.1016/j.diabres.2025.112196. 40268141 10.1016/j.diabres.2025.112196

[14] Turkish Endocrinology and Metabolism Association. Guide to diagnosis, treatment and follow-up of diabetes mellitus and its complications. Turkish, Endocrinology, and Metabolism Association; 2024. https://file.temd.org.tr/Uploads/publications/guides/documents/diabetesmellitus2024.pdf. Accessed: June 29, 2025.

[15] Liu NF, Brown AS, Younge MF, Guzman SJ, Close KL, Wood R. Stigma in people with type 1 or type 2 diabetes. Clin Diabetes. 2017;35(1):27–34. 10.2337/cd16-0020. 28144043 10.2337/cd16-0020 PMC5241772

[16] Felton BJ, Revenson TA. Coping with chronic illness: a study of illness controllability and the influence of coping strategies on psychological adjustment. J Consult Clin Psychol. 1984;52(3):343–53. 10.1037//0022-006x.52.3.343. 10.1037//0022-006x.52.3.343 6747054

[17] Alwatify SSD, Radhi MM. Diabetes self-management and its association with medication adherence in diabetic patients. Iran J War Public Health. 2025;17(1):17–22.

[18] Wang RH, Lin CC, Chen SY, Hsu HC, Huang CL. The impact of self-stigma, role strain, and diabetes distress on quality of life and glycemic control in women with diabetes: A 6-month prospective study. Biol Res Nurs. 2021;23(4):619–28. 10.1177/10998004211009606. 33874782 10.1177/10998004211009606

[19] Browne JL, Ventura AD, Mosely K, Speight J. Measuring the stigma surrounding type 2 diabetes: development and validation of the type 2 diabetes stigma assessment scale (DSAS-2). Diabetes Care. 2016;39(12):2141–8. 10.2337/dc16-0117. 27515964 10.2337/dc16-0117

[20] Inkaya B, Karadag E. Turkish validity and reliability study of type 2 diabetes stigma assessment scale. Turk J Med Sci. 2021;51(3):1302–6. 10.3906/sag-2006-255. 33486913 10.3906/sag-2006-255 PMC8283479

[21] Buyukkaya Besen D, Esen A. The adaptation of the acceptance of illness scale to the diabetic patients in Turkish society. TAF Prev Med Bull. 2011;10(2):155–64.

[22] Ozkan Tuncay F, Koçyiğit N. The relationship between perceived stigma and health-promoting self-care in adult patients with type 2 diabetes. J Diabetes Metab Disord. 2025;24(1):49–17. 10.1007/s40200-025-01565-0. 39845909 10.1007/s40200-025-01565-0 PMC11748663

[23] Can S, Cicek SC, Abant AB, Ankarali H. The effect of illness acceptance on diabetes self care activities in diabetic individuals. Int J Caring Sci. 2020;13(3):2191–200.

[24] Parviniannasab AM, Faramarzian Z, Hosseini SA, Hamidizadeh S, Bijani M. The effect of social support, diabetes management self-efficacy, and diabetes distress on resilience among patients with type 2 diabetes: A moderated mediation analysis. BMC Public Health. 2024;24(1):477. 10.1186/s12889-024-18022-x. 38360647 10.1186/s12889-024-18022-x PMC10868118

[25] Olesen K, Cleal B, Willaing I. Discrimination and stigma among people with type 2 diabetes in the workplace: prejudice against illness or obesity? Public Health. 2020;180:100–1. 10.1016/j.puhe.2019.11.009. 31881462 10.1016/j.puhe.2019.11.009

[26] Turen S, Yilmaz RA, Gundogdu S. The relationship with acceptance of illness and medication adherence in type 2 diabetes mellitus patients. Int J Caring Sci. 2021;14(3):1824.

[27] Zheng F, Liu S, Liu Y, Deng L. Effects of an outpatient diabetes self-management education on patients with type 2 diabetes in china: A randomized controlled trial. J Diabetes Res. 2019;2019:1073131. 10.1155/2019/1073131. 30800684 10.1155/2019/1073131 PMC6360047

[28] Aktan Bektaş N, Mercan Başpınar M, Başat O. Evaluation of the stigma level related to chronic disease in diabetes and hypertension patients. Jour Turk Fam Phy. 2023;14(1):3–16. 10.15511/tjtfp.23.00102.

[29] Cho SE, Kwon M, Kim SA. Influence of diabetes knowledge, self-stigma, and self-care behavior on quality of life in patients with diabetes. Healthc (Basel). 2022;10(10):1983. 10.3390/healthcare10101983. 10.3390/healthcare10101983 PMC9602474 36292430

[30] Kobos E, Serafin O, Kostrzewa-Zabłocka E, Stefanowicz-Bielska A. Social support and disease acceptance in patients with diabetic foot syndrome and their relationship with the metabolic control of the disease. J Clin Med. 2025;14(10):3412. 10.3390/jcm14103412. 40429406 10.3390/jcm14103412 PMC12112424

[31] Kara SN. The effect of education given to caregivers of Alzheimer’s patients enrolled in home health services on their knowledge and attitudes towards Alzheimer’s disease: A randomised controlled study [master’s thesis]. Ordu University; 2025.

[32] Li X, Wu L, Yun J, Sun Q. The status of stigma in patients with type 2 diabetes mellitus and its association with medication adherence and quality of life in china: A cross-sectional study. Med (Baltim). 2023;102(26):e34242. 10.1097/MD.0000000000034242. 10.1097/MD.0000000000034242 PMC10313242 37390244

[33] Joachim G, Acorn S. Stigma of visible and invisible chronic conditions. J Adv Nurs. 2000;32(1):243–8. 10.1046/j.1365-2648.2000.01466.x. 10886457 10.1046/j.1365-2648.2000.01466.x

[34] Jankowiak B, Kowalewska B, Krajewska-Kułak E, Khvorik DF, Niczyporuk W. Relationship between self-esteem and stigmatization in psoriasis patients. Postepy Dermatol Alergol. 2020;37(4):597–602. 10.5114/ada.2020.93242. 32994785 10.5114/ada.2020.93242 PMC7507155

[35] Ozyalcin B, Sanlier N. Evaluation of disease acceptance, depression, and quality of life in people with type 2 diabetes mellitus. Acta Endocrinol (Buchar). 2022;18(4):474–9. 10.4183/aeb.2022.474. 37152883 10.4183/aeb.2022.474 PMC10162829

[36] Rogon I, Kasprzak Z, Szcześniak Ł. Perceived quality of life and acceptance of illness in people with type 2 diabetes mellitus. Prz Menopauzalny. 2017;16(3):79–85. 10.5114/pm.2017.70583. 29507573 10.5114/pm.2017.70583 PMC5834920

[37] Krzemińska SA, Kostka AM. Acceptance of illness and quality of life in patients with type 2 diabetes. J Educ Health Sport. 2021;11(5):86–100. 10.12775/jehs.2021.11.05.009.

